# Hyperresistinemia in Obese Female Dogs With Mammary Carcinoma in Benign-Mixed Tumors and Its Correlation With Tumor Aggressiveness and Survival

**DOI:** 10.3389/fvets.2020.00509

**Published:** 2020-08-13

**Authors:** Bianca Oliveira Nicchio, Stella Maria Barrouin-Melo, Marilia Carneiro Machado, Carlos Humberto Vieira-Filho, Ferlando Lima Santos, Emanoel Ferreira Martins-Filho, Vivian Fernanda Barbosa, Thiago Doria Barral, Ricardo Wagner Portela, Karine Araújo Damasceno, Alessandra Estrela-Lima

**Affiliations:** ^1^School of Veterinary Medicine, Federal University of Bahia, Salvador, Brazil; ^2^Research Center on Mammary Oncology NPqOM/HOSPMEV/UFBA, Salvador, Brazil; ^3^Health Science Center, Federal University of the Recôncavo of Bahia, Santo Antônio de Jesus, Brazil; ^4^Laboratory of Immunology and Molecular Biology, Institute of Health Sciences, Federal University of Bahia, Salvador, Brazil; ^5^Laboratory of Experimental Pathology, Gonçalo Moniz Institute, Oswaldo Cruz Foundation, Salvador, Brazil

**Keywords:** adipocytokines, Ki-67, mammary cancer, survival, obesity, prognostic marker

## Abstract

Resistin is associated with metabolic, inflammatory, and neoplastic disorders, and is also considered a prognostic marker in human oncology. Canine mammary tumors have epidemiological, clinical, biological, and genetic characteristics similar to those of women and are proposed as a comparative study model. Here, we evaluate the serum levels of resistin in female dogs with or without mammary carcinoma in mixed tumors (CBMT) and its correlation with the proliferative potential of the tumor, obesity, and survival. Eighty dogs grouped according to the presence (50) or absence (30) of CBMT, reproductive status and body condition were assessed for weight, fat percentage, and canine body mass index. The characteristic of the proliferative potential of the tumor (Ki-67) was evaluated. Ki-67 levels (*p* = 0.024), staging (*p* = 0.004), and grade (*p* = 0.016) influenced the survival of the female dogs. Through a multifactorial analysis, it could be seen that the parameters proliferation index (Ki-67) (*p* = 0.044) and staging (*p* = 0.036) influenced the survival of the animals. Neutered and overweight dogs from the control and CBMT groups showed hyperresistinemia. Ki-67 expression and resistin levels in dogs with CBMT were higher in overweight dogs than in dogs with normal weight (*p* = 0.0001). The survival rate of dogs with CBMT, obese and with high levels of resistin (8,400 μg L^−1^) was lower when compared to those with lower levels of resistin. These results showed an important relationship between hyperresistinemia, tumor proliferative potential and excessive body fat, suggesting that resistin levels may act as an interesting prognostic marker in patients with CBMT.

## Introduction

Female dogs with natural mammary cancers have been considered a model for the comparative and translational study of breast cancer in women (1–4). Scientific evidence of the association between obesity and human breast cancer has been verified and described in several studies (5–8). Obese women with breast cancer also show a marked dysregulation of insulin signaling, which causes an imbalance in cell proliferation and differentiation, apoptosis, increased concentration of pro-inflammatory cytokines, with a consequent influence on adipocytokines (9) and C-reactive protein (CRP) levels (10).

Adipocytokines, such as resistin, are correlated with obesity, acting in the regulation of blood glucose, homeostasis, and insulin resistance (11). Some authors suggest that this relationship is under genetic control (12, 13), but the mechanism of expression, regulation, secretion, and circulation of resistin remain unclear (14). High concentrations of resistin caused by genetic or external factors, such as obesity and diet, can play an important role in the pathogenesis of some neoplasms, such as breast cancer (15). Although this correlation is not well-understood, it is known that resistin promotes epithelial and mesenchymal multiplication in the breast parenchyma, which are critical mechanisms for tumor development and metastasis (16). In addition, resistin concentrations are positively correlated with malignancy, tumor stage and size and presence of metastasis (16, 17), which makes this molecule a biomarker for breast tumor prognosis (18, 19).

In domestic animals, the origin of resistin has not yet been documented (20) and little is known about the pathophysiological mechanisms that involve resistin in neoplasms (21, 22). As already demonstrated in humans, dogs with a tendency to obesity that undergo neoplastic processes tend to have a higher expression of certain adipocytokines (23, 24). Mammary carcinoma in mixed benign tumors (CBMT) has variable malignancy potential and are the most frequent histological type found in canine mammary neoplasms (25–29). However, despite the high frequency of CBMT in the oncology routine, according to our knowledge, there are no studies on the possible participation of resistin in this tumor progression. Thus, the aim of this study was to investigate the serum concentration of resistin in dogs with CBMT and its relationship with obesity, tumor aggressiveness, and survival. We focused in CBMT with the objective to have a more reliable and specific answer, since different tumor histological types can influence on the variables studied herein, such as the prognosis.

## Materials and Methods

### Ethical Statement

The research procedures herein presented were approved by the Ethics Committee on the Use of Animals of the School of Veterinary Medicine of the Federal University of Bahia (protocol no. 05/2015). All procedures listed in this study were conducted according to the guidelines set by the Brazilian College of Animal Use on Experiments (COBEA). All animals were domiciliated and dog guardians were informed about the details of the research project, by means of a signed Free and Informed Consent Form.

### Animals and Clinical Evaluation

Eighty female dogs, treated at the Hospital-School of Veterinary Medicine of UFBA, Salvador, Brazil, from March 2016 to March 2018, with different body conditions and breeds were selected, with ages varying from 4 to 13 years. Of these, 50 dogs with mammary cancer and histopathological diagnosis of CBMT formed the experimental group. The control group consisted of 30 healthy dogs with no history of tumor. Inclusion criteria for the CBMT group included a histopathological diagnosis of CBMT and no other concomitant disease at the time of evaluation. Dogs presenting infectious diseases, such as leishmaniasis, babesiosis, ehrlichiosis, or endocrine pathologies, such as diabetes, hypothyroidism, and Cushing syndrome were excluded from the study. The use of anti-inflammatory medication 30 days before the mastectomy was also considered an exclusion criterion.

All dogs underwent a clinical examination for complete systemic evaluation, mammary neoplasm diagnosis, and classification of body condition. Female dogs were categorized within each group (CBMT and Control). With the objective to group the animals according to their body condition (adequate weight or overweight), an assessment of body condition was performed. The determination of body condition was based on two methods, the Canine Body Mass Index (BMIc) (30) and the Percentage of Body Fat (% GC) (31). BMIc was obtained using the formula BMIc = weight (kg)/(height in m)^2^. The ideal BMIc for medium-sized dogs (10–25 kg) was considered as being between 11.8 and 15 kg m^−^^2^ while overweight/obese dogs have a BMIc above 15 kg/m^2^. For small and large breeds, BMIc can be estimated by decreasing 10% and adding 20% to the BMIc of medium-sized animals, as determined by Muller et al. (30). For the examination of body composition, the percentage of body fat (%BF) was determined, which in animals with an ideal weight is between 16 and 25%, and in animals with overweight/obese is higher than 25%. According to Burkholder and Toll (31) the equation that defines the percentage of fat in female dogs is % CG = −1.7 (MP cm) + 0.93 (PA cm) + 5. In this equation, % GC stands for percentage of body fat, MP indicates the length of the right posterior limb, measured from the calcaneal tuberosity to half of the patellar ligament, and PA means perimeter of the abdomen. Animals with body mass index (BMIc) > 15.1 kg/m^2^ or body fat percentage (%BF) >25% were considered overweight/obese.

Preoperative clinical evaluation included peripheral blood sampling for hemogram, serum biochemical analyses, thoracic radiological examination (laterolateral right, laterolateral left, and ventral-dorsal), and total abdominal ultrasound to assess for metastasis. Clinical stage classification was performed based on tumor size (T), involvement of regional lymph nodes (N), and presence or absence of distant metastases (M), based on the TNM system (32). Macroscopic evaluation of inguinal and axillary lymph nodes was performed by palpation. Neoplastic involvement was confirmed by histopathological examination of lymph nodes following mastectomy.

### Mastectomy and CBMT Histopathological Characterization

All dogs were subjected to unilateral total mastectomy, with the removal of inguinal lymph nodes. Fragments of the affected mammary gland, including skin, and subcutaneous tissues, were fixed in phosphate-buffered 10% neutral formalin, and processed by the routine technique of paraffin embedding. Histological sections (4 μm) were stained by the hematoxylin-eosin (HE) method. In all cases, duplicate slides were prepared and analyzed by two veterinarian pathologists. Tumor samples were categorized according to the histopathological diagnosis following the World Health Organization (WHO) criteria, and complemented by the *Consensus regarding the diagnosis, prognosis, and treatment of canine mammary tumors: benign mixed tumors, carcinomas in mixed tumors and carcinosarcomas* (33). The histological grade of the tumor was described by the Nottingham system as modified by Elston and Ellis (34), which evaluates the percentage of tubule formation, nuclear pleomorphism and the mitotic index. The epithelial arrangement of the tumor was classified as tubular or papillary. In cases of association, that is, the tubulo-papillary arrangement, the predominant arrangement in histological sections was considered. The matrix was classified as myxoid, mixed (myxoid associated with chondroid or osteoid matrix) or myxoid associated with mature tissue (cartilage or bone trabeculae). The degree of desmoplasia was qualitatively classified as mild, moderate, or intense.

### Evaluation of Proliferation Index by Immunohistochemistry

Three micrometer tissue sections were cut from one representative block of each sample and collected onto glass slides. Tissue sections were deparaffinized in xylene, subjected to heat-induced antigen retrieval with an antigen retrieval solution, citrate buffer pH 6.0, in a water bath at 98°C for 20 min, and an endogenous peroxidase activity block was performed with 3% hydrogen peroxidase in methanol. Then, the anti-Ki-67 monoclonal antibody (Mib-1, Dako, 1:25) was added and incubated for 60 min at room temperature. The Novolink Polymer Detection System was then used in the immunohistochemical procedure (Ready to use, Leica Biosystems). Diaminobenzidine was used as a chromogen, and the sections were counterstained with Mayer's hematoxylin, dehydrated, and mounted in synthetic medium.

Negative controls were obtained by replacing the primary antibody with normal mouse serum. Canine mammary tumors previously known to express high levels of Ki-67 were used as positive controls. Ki-67 staining was considered positive when cell nuclei presented a diffuse nuclear staining pattern, and Ki-67 proliferative activity was assessed by image analysis through the determination of the percentage of positive cells among 1,000 tumor cells (proliferation index) (35). In the present study, the presence of more than 14% of stained nuclei were considered to classify the case as presenting a high rate of cell proliferation.

### Quantification of Serum Resistin

To evaluate the concentration of serum resistin (μg L^−1^), 3 mL of blood were collected from each dog during pre-surgical evaluation by jugular vein puncture in tubes without anticoagulant (VacuTainer). The samples were centrifuged for 5 min at 3,000 × g and sera separated in aliquots and stored in cryotubes at −80°C. Resistin concentrations (μg L^−1^) were then determined using an immunosorbent assay (ELISA – Biomatik, reference EKB01946) specific for the detection of canine resistin. The assay presents an intra-assay reproducibility of >90%, an inter-assay reproducibility of >88%, a minimum detectable resistin concentration of 10 pg/mL and a linearity of 94% of the expected concentration. All samples were assayed in duplicate and completely blinded to the clinical information. All procedures followed the manufacturer's instructions and quality control measurements were within the ranges recommended by the manufacturer.

### Monitoring and Survival

All dogs included in the survival rate analysis were suctioned to follow-ups including systematic clinical evaluation, radiological examinations, and biochemical and hematological analysis. The overall survival rate, expressed in days, was defined as the time between surgical excision of the primary tumor and the date of death or end of follow-up. Dogs that died during the follow-up period were necropsied at the Veterinary Pathology Laboratory to determine the cause of death and to assess for possible metastasis.

### Performance Indexes of Resistin Levels

The receiver operating characteristic curve (ROC curve) (36) was used to select the best cut-off value for resistin serum concentrations with the objective to discriminate distinct evolution to death or survival. The performance analysis included the global accuracy analysis, which was evaluated by the area under the ROC curve (AUC) as proposed by Swets (37). The formulae used were: Co-positivity (Co-pos) = [true positives/(true positive samples + false negative samples)] × 100; Co-negativity (Co-neg) = [true negatives/(true negative samples + false positive samples) × 100; Positive predictive value (PPV) = (true positive samples/total of positive samples) × 100; Negative predictive value (NPV) = (true negative samples/total of negative samples) × 100; Positive Likelihood ratio (LR+) = Co-positivity/(1 - Co-negativity); Negative Likelihood ratio (LR–) = (1 - Co-positivity) /Co-negativity).

### Statistical Analysis

The data were grouped as follows: tumor size (5 < or ≥5 cm), clinical staging (I–II or III–IV), histological grade (I or II), reproductive status (neutered or not), Ki-67 (≤ 14 and >14%), BMIc (≤ 15.1 and >15.1 Kg/m^2^), BF% (≤ 25 and >25%), and resistin serum levels (≤ 8,400 and >8,400 μg mL^−1^). For statistical analysis, the chi-square-Test was used to compare the relevance between category variables of breast cancer. The survival rate was classified as low when the survival values were <365 days. Initially, the Kolmogorov-Smirnov test was applied to evaluate the normality of data distribution. Student's *t*-tests were used for the variables with normal distribution. The Mann-Whitney test was used for the variables without normal distribution. Possible correlations were investigated by the Spearman test. Survival curves were generated by the Kaplan-Meier estimation method and compared by Log-rank Mantel-Cox or Cox proportional hazards tests in univariate or multivariate analysis, respectively. The analyses were performed using Prism 5.0 software (GraphPad, San Diego, CA, USA), SPSS 17 (SPSS Inc., Chicago, IL, USA), and MedCalc for Windows version 15.0 (MedCalc Software, Ostend, Belgium).

The statistical analysis of body and reproductive status profiles was performed in two steps. In the first step, analyses were performed using the GLINMIX procedure in SAS software (version 9.2), where body status, presence or absence of CBMT, reproductive status (neutered or not), and the interaction between these variables were considered fixed effects in the statistical model. The following continuous probability distributions were tested for each variable: exponential, log-normal, gamma, Weibull, t-distribution, inverse Gaussian, and normal. The criteria for obtaining the best fit for these distributions were the maximum likelihood and the relation between chi-square and degrees of freedom, which were considered better when they were closer to 1. For the comparison between the maximum likelihood means, the *p*-value of each comparison was used as conclusive. All analyses were conducted using 0.05 as the critical level of probability for type I error.

## Results

### Clinical-Pathological and Corporal Parameters of Female Dogs With CBMT

**Figure 1:**
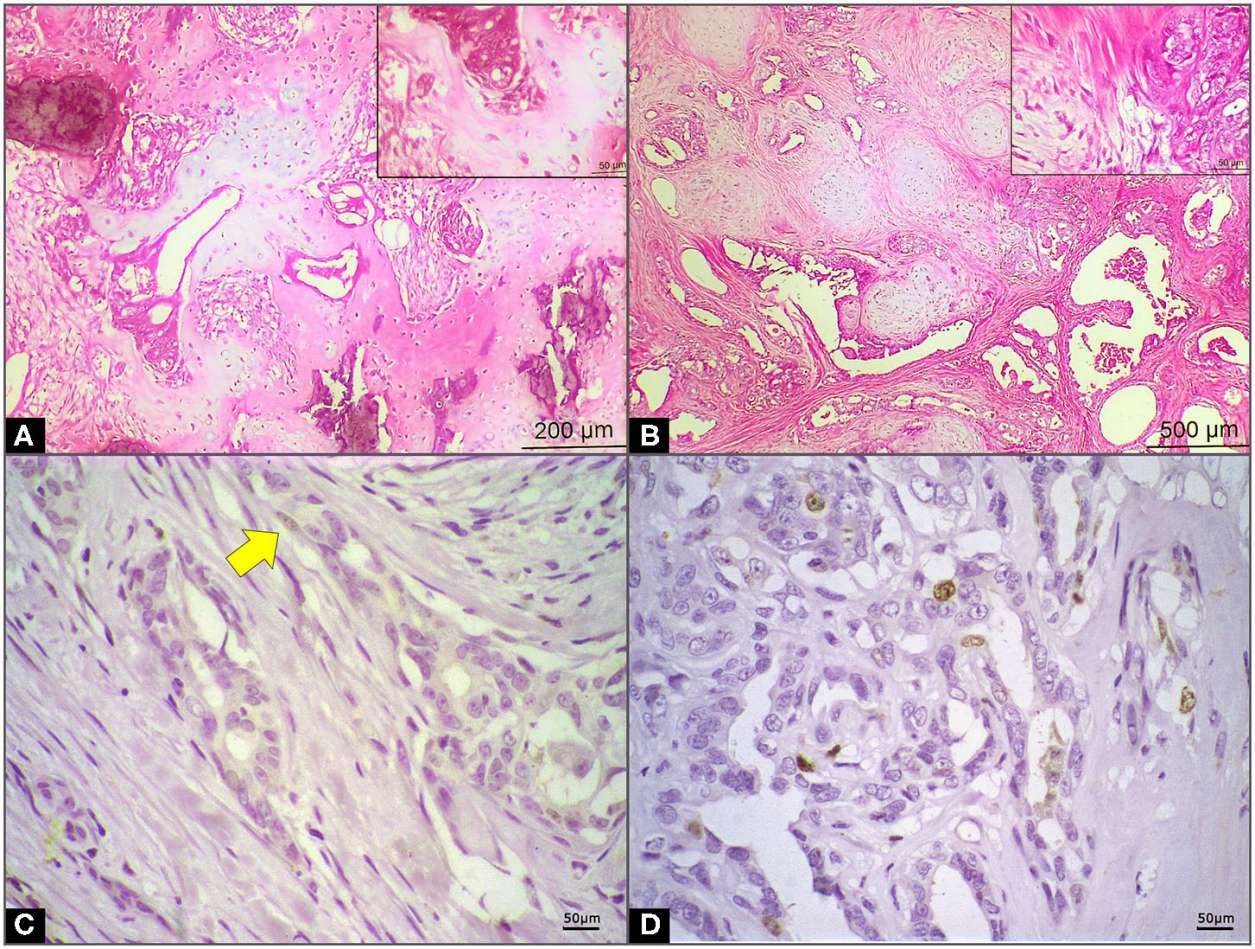
Microscopical and immunohistochemistry features in mammary carcinoma in mixed tumor (CBMT) of female dogs. **(A)** CBMT grade I with epithelial portion characterized by irregular tubules coated by discrete pleomorphic cells with scarce cytoplasm, small nuclei, and small foci of infiltrative growth. The myoepithelial cells associated with formation of cartilage and bone trabeculae with foci of mineralization can be observed (highlighted)—HE 10x/40x. **(B)** CBMT grade II with epithelial portion characterized by irregular papillae and tubules coated by moderately pleomorphic cell layers with sparse cytoplasm, medium, round or ovoid nuclei, vesicular with evident nucleolus. The myoepithelial cells proliferation with myxoid matrix areas can be observed—HE 4x/40x (highlighted). **(C)** Ki-67 expression in CBMT of female dog in normal body condition (arrow) **(D)** Ki-67 expression in CBMT of female obese dogs.

Table 1 shows the quantitative and qualitative clinical characteristics of the healthy dogs from the control group and of the dogs from the CBMT group. The clinical staging evaluation of the dogs from the CBMT group regarding body condition showed that a larger number of overweight dogs had tumor sizes with <5 cm when compared to the dogs with normal weight. All of the CBMTs that were observed in this study presented the classifications Grade I (low) or II (intermediate) (Figures 1A,B).

**Table 1:** General characteristics, clinical and pathological aspects, and body condition of female dogs with mixed tumor carcinoma (CBMT) and healthy female dogs included in this study.

**QUANTITATIVE PARAMETERS**						
	**Healthy control (*****n*** **=** **30)**			**CBMT (*****n*** **=** **50)**		
	**Overweight/obese (*****n*** **=** **15)**	**Normal (*****n*** **=** **15)**	***P*****-value**	**Obese/overweight (*****n*** **=** **25)**	**Normal (*****n*** **=** **25)**	***p*****-value**
Age	8.9 ± 0.67^a^	7.3 ± 0.66^a^	0.122	9.08 ± 0.38^a^	8.68 ± 0.41^a^	0.4821
%BF	41.33 ± 3.31^a^	15.8 ± 0.87^a^	<0.0001^*^	31.58 ± 2.66^b^	14.06 ± 1.45^a^	<0.0001^*^
BMIc	27.71 ± 1.06^a^	11.36 ± 0.10^a^	<0.0001^*^	26.85 ± 0.83^a^	9.37 ± 0.50^b^	<0.0001^*^
Ki67				55.08 ± 5.55	9.83 ± 0.96	<0.0001^*^
Not neutered				51.92 ± 6.24	8.26 ± 1.05	<0.0001^*^
Neutered				63.21 ± 11.89	12.18 ± 1.62	0.0001^*^
Resistin	9,273 ± 238^a^	3,349 ± 284^a^	<0.001^*^	15,584 ± 1,051^b^	5,780 ± 872^b^	<0.0001^*^
Not neutered	9,330 ± 736^a^	3,065 ± 407^a^	<0.0001	14,968 ± 1,185^b^	5,362 ± 770^a^	<0.0001^*^
Neutered	9,253 ± 222^a^	3,598 ± 398^a^	<0.0001	17,168 ± 2,229^b^	6,407 ± 1,902^a^	0.0023^*^
Weight	24.93 ± 3.19^a^	13.9 ± 1.14^a^	0.003^*^	12.36 ± 1.86^b^	6.2 ± 0.37^b^	0.0018^*^
Not neutered	26.25 ± 6.77^a^	15.34 ± 2.30^a^	0.0928	8.71 ± 0.88^b^	6.28 ± 0.56^b^	0.0314^*^
Neutered	24.45 ± 3.79^a^	12.64 ± 0.64^a^	0.0426^*^	21.24 ± 4.71^a^	6.06 ± 0.44^b^	0.2527
**QUALITATIVE PARAMETERS**						
	**Healthy control (*****n*** **=** **30)**			**CBMT (*****n*** **=** **50)**		
	**Obese/overweight (*****n*** **=** **15)**	**Normal (*****n*** **=** **15)**		**Obese/overweight (*****n*** **=** **25)**	**Normal (*****n*** **=** **25)**	
Small breeds	33.3% (5/15)	40.0% (6/15)		64.0% (16/25)	56.0% (14/25)	
Neutered	60.0% (3/5)	33.3% (2/6)		25.0% (4/16)	35.7% (5/14)	
Non-neutered	40.0% (2/5)	66.7% (4/6)		75.0% (12/16)	64.3% (9/14)	
Medium/large breeds	66.7% (10/15)	60.0% (9/15)		36.0% (9/25)	44.0% (11/25)	
Neutered	80.0% (8/10)	66.7% (6/9)		33.3% (3/9)	45.5% (5/11)	
Non-neutered	20.0% (2/10)	33.3% (3/9)		66.7% (6/9)	54.5% (6/11)	
			**CBMT**			
			**Body condition**			
			**Normal (*****n*** **=** **25)**		**Obese/overweight (*****n*** **=25)**	
			**Not neutered (*****n*** **=** **15)**	**Neutered (*****n*** **=** **10)**	**Not neutered (*****n*** **=** **18)**	**Neutered (*****n*** **=** **07)**
Tumor size	<5 cm		66.7% (10/15)	40.0% (4/10)	50.0% (9/18)	28.6% (2/7)
	≥ 5 cm		33.3% (5/15)	60.0% (6/10)	50.0% (9/18)	71.4% (5/7)
Clinical staging	I		26.7% (4/15)	0.0% (0/10)	22.2% (4/18)	14.3% (1/7)
	II		33.3% (5/15)	30.0% (3/10)	27.8% (5/18)	14.3% (1/7)
	III		20.0% (3/15)	60.0% (6/10)	38.9% (7/18)	14.3% (1/7)
	IV		20.0% (3/15)	10.0% (1/10)	11.1% (2/18)	57.1% (4/7)
Histological	I		60.0% (9/15)	80.0% (8/10)	50.0% (9/18)	14.3% (1/7)
Grade	II		40.0% (6/15)	20.0% (2/10)	50.0% (9/18)	85.7% (6/7)
CBMT	Tubular		66.7% (10/15)	80.0% (8/10)	27.8% (5/18)	12.5% (1/7)
Cell arrangement	Papillary		33.3% (5/15)	20.0% (2/10)	72.2% (13/18)	87.5% (6/7)
CMT	Myxoid		66.7% (10/15)	70.0% (7/10)	50.0% (9/15)	57.1% (4/7)
Matrix arrangement	Mixed (Myxoid + condróide ou osteoid)		20.0% (3/15)	20.0% (2/3)	27.8% (5/15)	14.3% (1/7)
	Myxoid matrix + Mature tissue (cartilage or bone)		13.3% (2/15)	10.0% (1/3)	22.2% (4/15)	28.6% (2/7)
CBMT	Discret		40.0% (6/15)	50.0% (5/10)	33.3% (6/18)	28.6% (2/7)
Stroma/desmoplasia	Moderate		40.0% (6/15)	10.0% (4/10)	38.9% (7/18)	28.6% (2/7)
	Intense		20.0% (3/15)	10.0% (1/10)	27.8% (5/18)	42.9% (3/7)
Outcome	Live		86.7% (13/15)	80.0% (8/10)	83.3% (15/18)	28.6% (2/7)
	Death		13.3% (2/15)	20.0% (2/10)	16.7% (3/18)	71.4% (5/7)

* *Stands for significant differences at the Unpaired t-test statistical test with p < 0.05. Different superscript letters stand for significant differences between groups at the Unpaired t-test statistical test with p < 0.05*.

When comparing the variables weight, BMIc and BF%, they were significantly higher in the subgroups of animals with overweight/obese body condition, regardless of the considered group (control or CBMT) (Table 1). The analysis of the relationship between reproductive status and body weight showed in the control group that the neutered dogs had a significantly higher body weight (*p* = 0.0426), while in the CBMT group non-neutered dogs had a significantly higher body weight (*p* = 0.0012) (Table 1).

### Proliferation Index (Ki-67)

**Figure 2:**
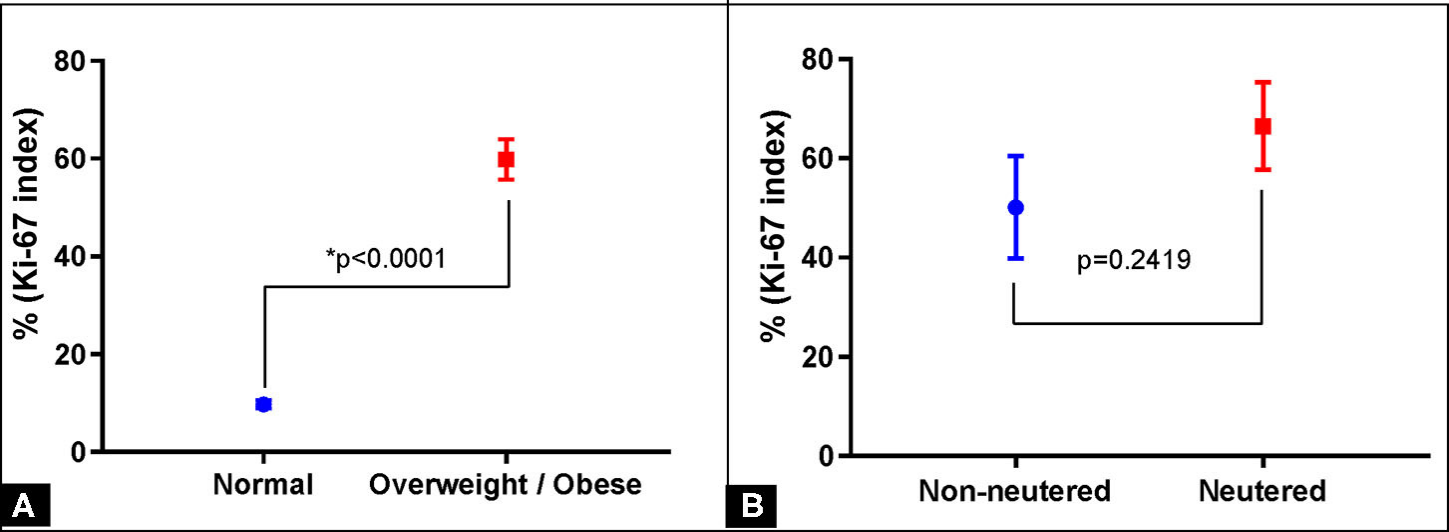
Proliferation index (Ki-67) in mammary carcinoma in mixed benign tumor (CBMT) of female dogs and body condition relationship. **(A)** Correlation between the Ki-67 index and body condition (normal or overweight/obese). **(B)** Correlation between the Ki-67 index and the reproductive status (not-neutered and neutered).

The Ki-67 count varied from 9.83 ± 0.96 and 55.08 ± 5.55 in the group of dogs with normal body conditions and in the group of overweight/obese dogs in the CBMT group, respectively (Table 1) (Figures 1C,D). Ki-67 values in the CBMT group were significantly higher in the subgroup with overweight/obese (*p* < 0.0001) than in the subgroup with normal body condition (Figure 2A). It has not been demonstrated that castration has an influence on Ki-67 values in the CBMT group (Figure 2B). Among overweight dogs, the proliferation rate was higher in the group with higher serum concentrations of resistin. The correlation coefficient between the Ki-67 index and serum resistin values in dogs with overweight CBMT was 0.4263 with a *p*-value of 0.0336.

### Quantification of Resistin in Serum

**Figure 3:**
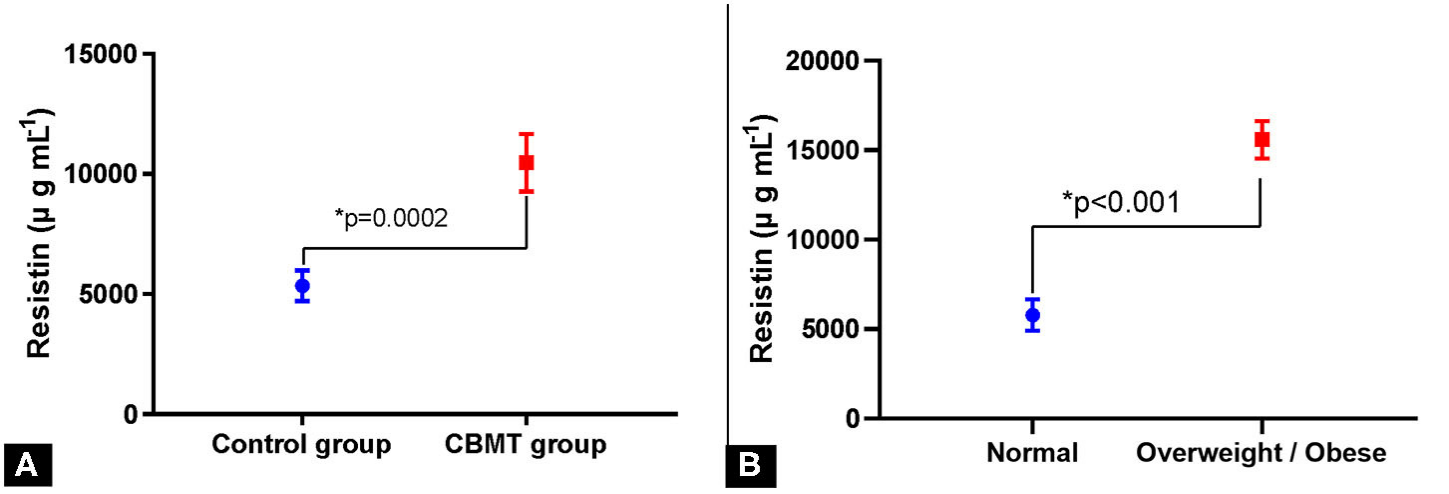
Resistin levels and body condition relationship. **(A)** Serum levels of resistin in female dogs with and without mammary carcinoma in benign mixed tumor (CBMT). **(B)** Serum resistin concentrations in female dogs with mammary carcinoma in benign mixed tumor and presenting normal and overweight/obese body condition.

The mean serum resistin concentrations were 6,311 ± 579.4 μg mL^−1^ and 10,682 ± 973.3 μg mL^−1^ in the healthy group and in the CBMT group, respectively. Resistin was significantly higher in the CBMT group (*p* = 0.002) than in the healthy control group (Figure 3A). In the CBMT group, overweight dogs had a higher mean serum resistin (15,584 ± 1,051 μg mL^−1^) when compared to dogs with normal weight (5,780 ± 872.3 μg mL^−1^) (*p* < 0.0001) (Figure 3B). Castration did not influence serum resistin levels in animals from the healthy group or from the CBMT group.

**Figure 4:**
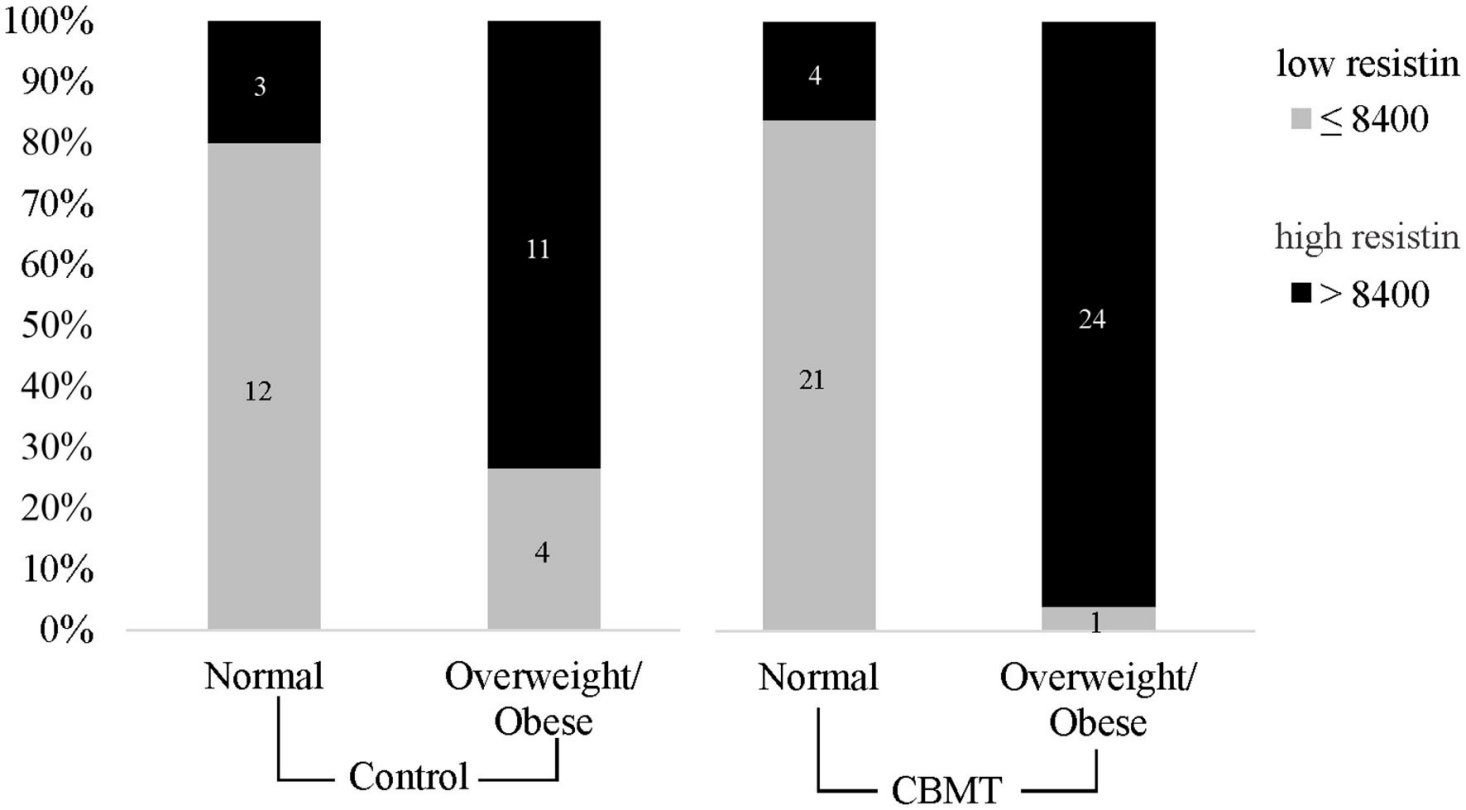
Hyperresistinemia is associated with body condition. A cutoff was established to distinguish high and low levels of resistin and CBMT animals were divided by their body condition.

A chi-square test was done to verify the relationship between resistin levels and body condition, both in the control group and in CBMT group. In the control group the relationship between these two variables was significant (*p* = 0.0034). The evaluation showed 11 truly positive dogs (overweight/obese and high resistin levels), 12 truly negative dogs (normal body condition and low resistin), 04 false positive animals (overweight/obese and low resistin), and 03 false negatives (normal body condition and high resistin). The overweight/obese animals presented a low probability to present low resistin (PPV = 0.73, IC 95% 0.48–0.89; NPV = 0.80, IC 95% 0.55–0.93). In the CBMT group, the relationship body condition/resistin level was also significant (*p* < 0.0001), and it were identified 24 truly positive dogs (overweight/obese and high resistin), 21 truly negative dogs (normal body condition and low resistin), 01 false positive (overweight/obese and low resistin), and 04 false negatives (normal body condition and high resistin levels). The overweight/obese animals presented a lower probability to present low resistin levels (PPV = 0.95, IC 95% 0.78–0.99; NPV = 0.86, IC 95% 0.69–0.94) (Figure 4).

### Performance Indexes of Resistin

**Figure 5:**
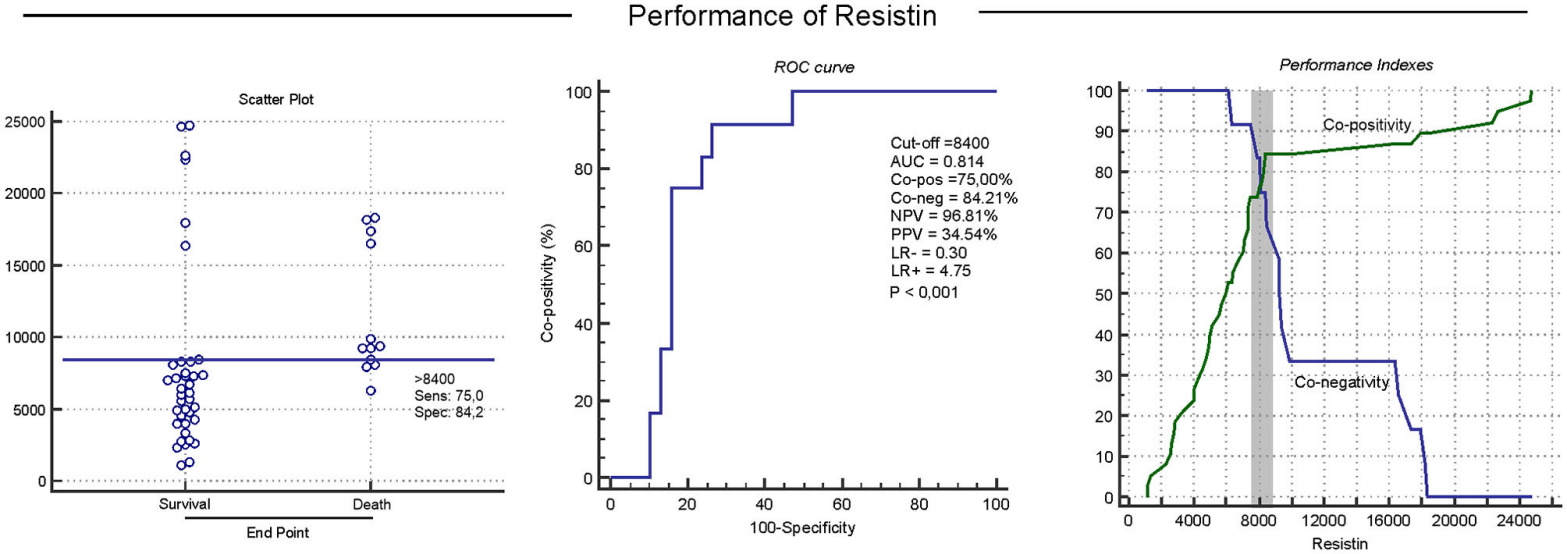
Serum resistin concentrations predict disease progression in relation to the survival or death of female dogs carrying CBMT. This situation was analyzed through performance indices, including segregation in scatter plots, ROC (Receiver Operating Characteristic) curve indices, Area Under the Curve/global precision (AUC), Co- positivity (Co-pos), Co-negativity (Co-neg), Positive and negative predictive values (NPV and PPV), as well as negative and positive Likelihood Ratio (LR– and LR+) Performance indices suggested that serum resistin concentrations >8,400 are useful biomarkers to predict survival in female dogs with CBMT.

The analysis of the Kaplan-Meier survival curves further validates the previous findings, demonstrating that animals with a serum resistin concentration >8,400 had a significantly lower survival rate. The additional correlation analysis also corroborates these results (Figure 5). The performance analysis chose the concentration of resistin of >8,400 as an edge to segregate, with very good rates, the result of survival or death at CBMT.

### Comparison of Survival Curves

**Figure 6:**
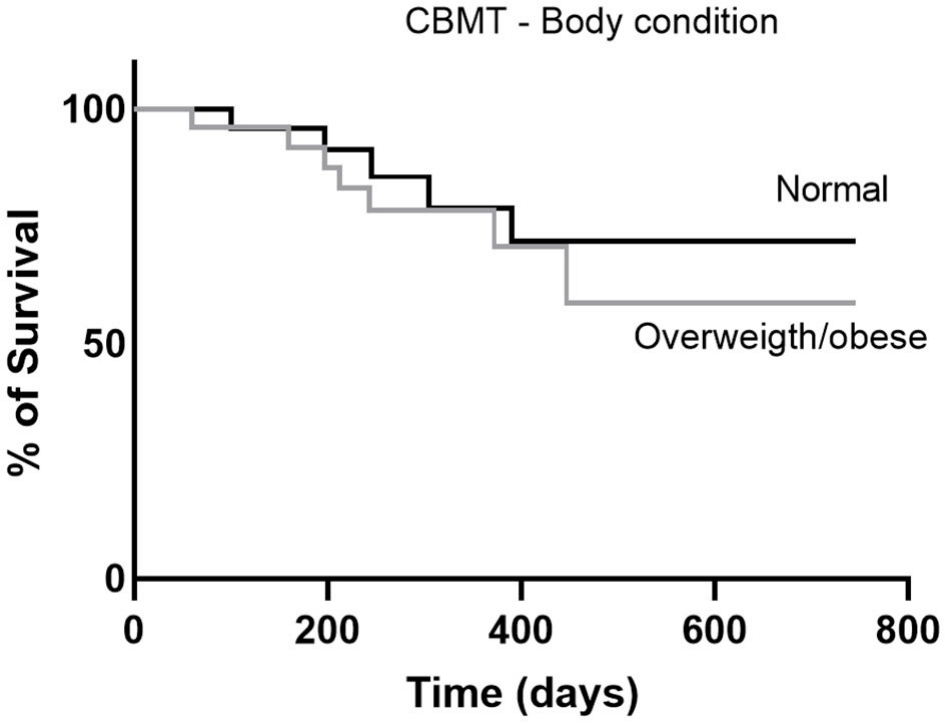
Survival curves of dogs with CBMT and with normal body condition or overweight/obese. The curves were constructed using the Kaplan-Meier method and compared by the log-rank test.

**Figure 7:**
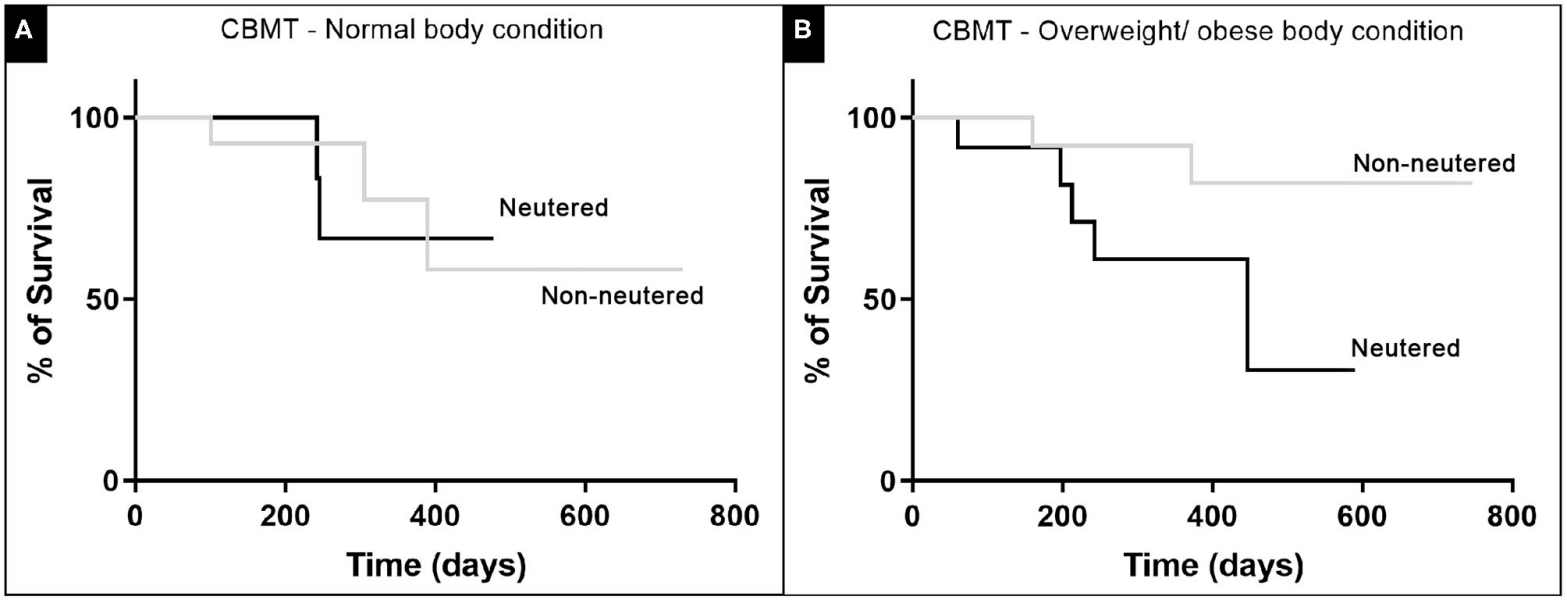
The influence of the reproductive status in CBMT female dog post-operative survival. The curves included **(A)** normal weight and **(B)** overweight/obese dogs. No significant statistical difference could be found for both comparisons, using the long-rank test and with *p* < 0.05.

**Figure 8:**
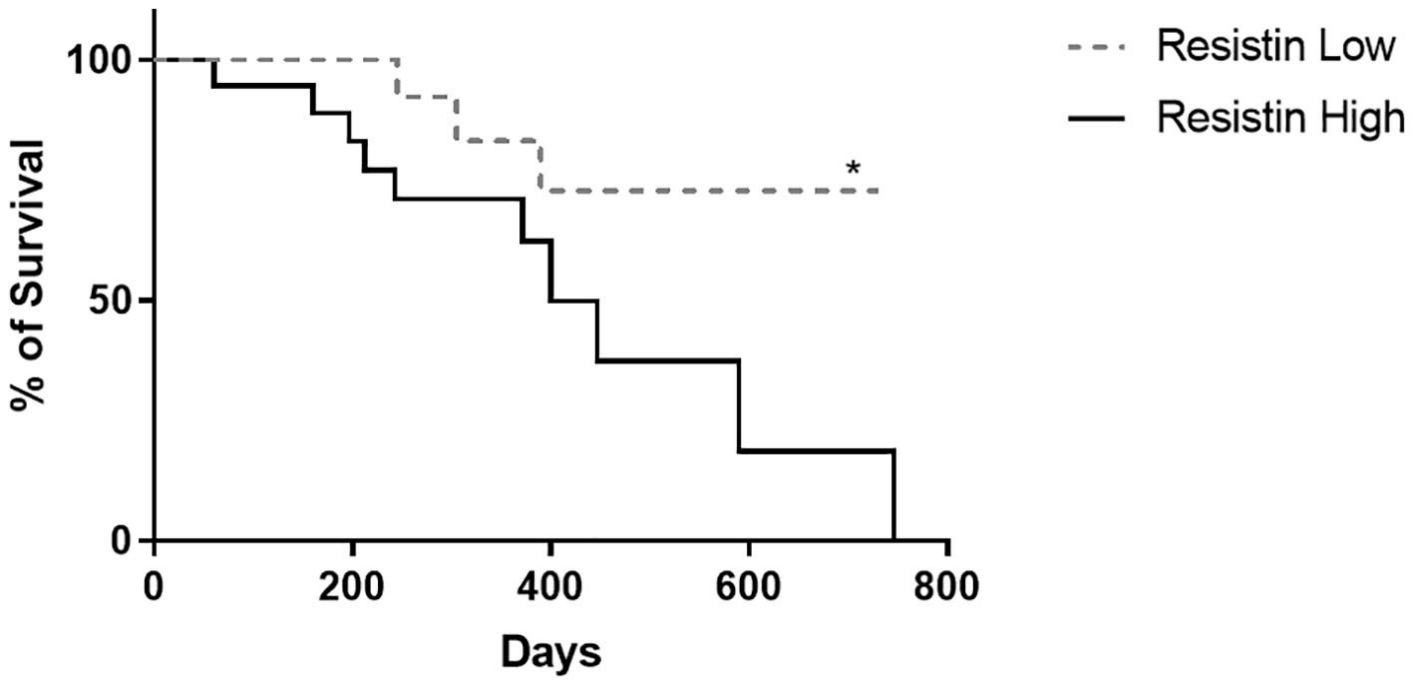
Survival curves of CBMT female dogs according to its serum resistin levels. (^*^) express a significant statistical difference among the curves by the log-rank test with *p* < 0.05.

The minimum survival after surgery was 60 days, attributed to an obese female dog, who died of respiratory failure. Histopathological evaluation of sections of the lung parenchyma revealed multiple micrometastasis. The maximum survival period was 730 days after mastectomy, attributed to a normal weight dog that is still being monitored. There were no statistically significant differences between the survival curves of CBMT dogs with normal weight and obese CBMT dogs, although a higher number of deaths was observed in the last group (*n* = 7/25; 28%, *p* = 0.5822, HR 4.152 and IC 95% 1.106–15.590) (Figure 6). The reproductive status did not influence the survival of dogs with normal body condition (*p* = 0.8630, HR 5.696, and IC 95% 1.591–20.394) (Figure 7A) or overweigh/obese (*p* = 0.0622) (Figure 7B), despite a higher number of deaths having been observed in the group of overweight/obese and neutered dogs (*n* = 5/7, 71.43%) (Tables 1, 2). On the other hand, there were significant differences between the survival curve of CBMT dogs with lower levels of resistin (≤ 8,400 μg L^−1^) and the survival curve of animals presenting higher levels of resistin (>8,400 μg L^−1^) (*p* = 0.0409, HR 3.275 and IC 95% 1.050–10.21). The highest number of deaths (*n* =10/28, 35.71%) occurred among dogs that presented hyperresistinemia (Figure 8).

**Table 2:** Overall survival times (median survival, median of subgroup, minimum, maximum, and 95% CI of median) of female dogs with mixed tumor carcinoma normal and overweight/obese body conditions.

	**Median survival (days)**	**Median sub group (days)**	**Minimum**	**Maximum**	**95% CI of median**	
					**Lower confidence limit**	**Upper confidence limit**
**CBMT and normal body condition**						
Not neutered	Undefined	259	100	730	176	400
Neutered	Undefined	242	123	478	134	450
**CBMT and overweight**/**obese**						
Not neutered	Undefined	400	160	746	250	600
Neutered	447	252	60	590	183	390

### Univariate and Multivariate Analysis

Ki-67, resistin, %BF, BMIc, and reproductive condition displayed a significant association with mortality. As revealed by the multivariate analysis, histological grade (*p* = 0.0001), Ki-67 (*p* = 0.0001), and BMIc (*p* = 0.014) remained as independent prognostic factors of mortality in the final model (Table 3). Additional analysis demonstrated significant association between %BF (*p* < 0.0001), BMIc (*p* = 0.0018), and Ki-67 (*p* < 0.0001) and distinct intervals of levels of resistin (Table 4).

**Table 3:** Associations between mortality and clinical-pathological parameters, analyzed by univariate and multivariate methods.

**Parameters**	**Univariate analysis**		**Multivariate analysis**	
	**Rho Spearman's (95% CI)**	***p*-value**	**Odds ratio (95% CI)**	***p*-value**
Tumor size ≥5 cm	0.080 (−0.211–0.358)	0.581	0.130 (0.004–4.558)	0.261
Clinical stage III–IV	0.097 (−0.195–0.372)	0.505	10.126 (0.235–435.597)	0.228
Histological grade (II)	0.244 (−0.045–0.496)	0.087	5.569 (1.026–30.2213)	0.015
Ki-67 ≥ 14%	0.694 (0.508–0.817)	0.0001	0.967 (0.935–0.999)	0.043
Resistin >8,400	0.866 (0.770–0.923)	0.0001	2.3873 (0.433–13.169)	0.046
BF% >25%	0.634 (0.425–0.779)	0.0001	1.0355 (0.903–1.188)	0.619
BMIc >15.1 Kg/m^2^	0.757 (0.601–0.858)	0.0001	2.0696 (1.107–3.868)	0.023
Reproductive condition (neutered)	0.926 (−0.274–0.298)	0.014	1.3871 (0.293–6.557)	0.995

*BF%, % of body fat; BMIc, canine body mass index*.

**Table 4:** Association between clinical-pathological parameters and distinct intervals of serum resistin concentrations.

**Parameters**		**Resistin**		***p***	**(Odds ratio; 95% CI)**
		**≤8,400**	**>8,400**		
BF%	≤ 25%	23	0	<0.0001^*^	(∞; 48.25–∞)
	>25%	1	18		
BMIc	≤ 15.1 Kg/m^2^	23	15	0.0022^*^	(16.87; 2.229–188.5)
	>15.1 Kg/m^2^	1	11		
Size	<5 cm	13	12	0.7775	(1.379; 0.4489–4.449)
	≥5 cm	11	14		
Clinical stage	l–II	12	11	0.7769	(1.364; 0.4397–4.419)
	III–IV	12	15		
Histological grade	I	16	11	0.0874	(2.727; 0.8640–8.240)
	II	8	15		
Ki-67	<14%	24	4	<0.0001^*^	(60.00; 9.803–291.3)
	≥14%	2	20		

* *Stands for significant differences at the chi-square test considering p < 0.05. BF%, % of body fat; BMIc, canine body mass index*.

## Discussion

Adipocytokines have been widely studied because they participate in mechanisms of cell proliferation, adhesion, and invasion, thus playing an important role in the development and progression of breast tumors (17, 18). However, little is known about the participation of resistin in mammary tumors in dogs. Dogs are commonly described as animal models for human breast cancer study, based on clinicopathologic similarities between canine and human mammary carcinomas (27). The present study aimed to evaluate the serum concentration of resistin in female dogs with CBMT, the most frequent histological type of breast tumor in this species, as well as its possible correlation with body condition, tumor aggressiveness, and survival rate (28, 29, 38, 39).

In the present study, there were dogs with body weight, BMIc, and %BF above or in the normal range in the healthy and in the CBMT groups. As a group, dogs with CBMT had lower mean values of weight, %BF, and BMIc when compared to dogs with normal body conditions. These findings can be related to the predominance of small dogs in the CBMT group (Table 1). However, within the CBMT group, the mean values of weight, %BF, and BMIc indexes of the obese dogs were up to 2.5 times greater than the same indexes in the dogs with CBMT and normal body condition, while in the healthy group, the difference between these indexes among obese compared to normal weight dogs was of 1.78 times. It should be emphasized that there is no standardized classification for measuring obesity in dogs, since the domestic dog exhibits a large variation of body sizes and conformations. Currently, the widely used methods for the diagnosis of obesity consider the body condition score (40), the morphometric analysis with determination of %BF (31), and BMIc (30). In the present study, we adopted the general term “body condition,” considering the weight, %BF, and BMIc values together, since there were no lean female dogs (weight, %BF, and BMIc below normal) in the groups studied.

The differences in body condition observed between neutered and non-neutered dogs, both in the CBMT and healthy control groups, confirm literature findings that neutered dogs are more predisposed to gain weight compared to non-neutered dogs (41). The excess body fat in neutered dogs occurs due to the reduction of the metabolic rate (42), especially in females, which naturally have lower basal metabolism than males (43). The results of the present study indicated a risk of death three to five times higher in female dogs with CBMT, and this situation was correlated with overweight and castration, respectively. However, castration is considered an essential measure when it comes to population control and is also considered an important protective measure against mammary carcinogenesis in female dogs, as it results in a decrease in circulating levels of estrogen and progesterone (44, 45). However, in all situations, castration must be associated with regular physical activity and a balanced diet to avoid undesirable consequences of obesity (42). The number of meals per day, the amount of food offered per meal and the provision of snacks, for example, are dietary practices involved in weight gain that need to be monitored (46).

Obese women and female dogs are more frequently affected by mammary tumors when compared to females with normal body conditions (24, 47–49). However, in both human and veterinary medicine, the mechanisms that lead to this occurrence are not fully understood (24, 50, 51). Obese women with mammary cancer also show a marked deregulation of insulin signaling, which causes an imbalance in cell proliferation, differentiation, apoptosis, altered expression of adipocytokines (9) and C-reactive protein (CRP) (10). Leptin and adiponectin are the most studied adipocytokines (20, 52, 53), but there are others important adipocytokines, such as IL-6, TNF-α, lipocalin 2, IL-8, apelin, and visfatin (51, 54). Adiponectin is a polypeptide hormone characterized by anti-inflammatory and anti-atherogenic activities, in addition to playing a key role in the metabolism of carbohydrates and lipids (51). Leptin and resistin present a similar behavior and can be found in high levels in conditions such as obesity (55) and breast cancer (49).

In the present study, a higher serum resistin concentration was observed in the group of female dogs with mammary carcinoma (CBMT) when compared to the group of healthy dogs. When segregated by body condition, it was found that obese female dogs, regardless of the group (Control or CBMT), had a serum resistin concentration ~3 times higher compared to female dogs with normal body condition. Finally, when evaluated in terms of reproductive status (neutered or not), a similar profile was observed, with neutered female dogs showing a higher concentration of resistin, regardless of the group (Control or CBMT). When dogs with CBMT were segregated based on the serum concentration of resistin, the animals with higher resistin levels presented lower survival and were mostly obese, while the majority of dogs with lower serum resistin presented higher survival rates and also had normal weight. The overweight dogs in the CBMT group had larger tumors and most of them with a higher histological grade. In addition, this group of overweight dogs also had a significantly higher serum concentration of resistin.

These results in dogs corroborate the findings described for humans, which indicate hyperresistinemia in overweight women with breast cancer (16, 51, 56–58). However, some studies have shown high values of serum resistin in women with breast cancer independent of BMI (49, 59), different from what was observed in the present study, in which obesity was a determining factor for hyperresistinemia, which is enhanced in the presence of the tumor.

The knowledge about resistin and its relationship with obesity and cancer is still scarce. Some authors describe that this relationship is under genetic control in different populations (12, 13), but the mechanism of expression, regulation, secretion, and circulation of resistin remains uncertain (14). High concentrations of resistin caused by genetic or external factors, such as obesity and diet, can play an important role in the pathogenesis of some neoplasms, such as breast cancer, for example (15). Glucose dysmetabolism, insulin resistance, and changes in adipokines secretion (resistin in particular) may be involved in the development and progression of breast cancer in overweight/obese pre- and postmenopausal women (11, 49). Although this correlation is not well-understood, it is known that resistin promotes epithelial and mesenchymal multiplication in the breast parenchyma, which are critical mechanisms for tumor development and metastasis (16), and also can be related to cancer associated chronic inflammation (59). There is a possibility that there are some mechanisms related to this process in patients with breast cancer, like upregulation of proinflammatory cytokines via the NF-κB pathway, an important component of cancer-promoting machinery (60). In addition, the higher the resistin concentration, the worse the malignancy, tumor stage, size, and presence of metastasis (16, 17), what justifies why it is considered a promising biomarker for breast tumor prognosis (18, 19, 61).

The data obtained in the present study indicate that obesity in female dogs was also associated with the malignancy potential of tumors, as evidenced by a pronounced cell proliferation, since a larger number of Ki-67 marked cells was observed in the group of obese dogs, the same group that presented higher concentrations of resistin and a larger number of cases with histopathological graduation II. Similarly, in the analysis of the tumor infiltrative potential in obese postmenopausal women with breast neoplasia, an association between obesity and potential for malignancy by Ki-67 also was also found (62). A proliferative potential is significantly higher in malignant lesions compared to benign ones (63). The obesity factor, by itself, is indicated as a triggering condition for malignant neoplasms in humans (64). In addition, adipocytokines with angiogenic characteristics are secreted by adipocytes (65), such as resistin (66). Although the mechanism by which obesity may lead to an increase in proliferative index is poorly understood, the very deregulation of angiogenic adipocytokine synthesis is believed to be an important factor (63). The present study provides evidence that hyperresistinemia, in addition to a higher BMIc, can promote a proportional increase in the tumor cell proliferation rate, which justifies the worse prognosis found in these cases.

The isolated comparison of the survival curves found for overweight dogs and dogs with normal body condition did not reveal significant differences. This result differs from those described in human and veterinary literature, which indicates lower survival for obese individuals with breast cancer (50, 51). In an evaluation of women with breast neoplasms, a lower survival rate, in addition to a worse prognostic factor was observed in females with higher body weights (66), while a significantly higher proportion of undifferentiated tumors (grade III) in obese dogs, with consequent lower survival, was observed in another study (24). In fact, the greater number of deaths of obese dogs in the CBMT group combined with the higher values of serum resistin in the present study is relevant, since it corresponds to literature data showing a lower life expectancy and prognosis in women under equivalent conditions (16, 17). The results herein obtained with the univariate and multivariate analyses revealed that, as observed in women (50, 51), there was a positive association between hyperresistinemia, BMIc, and %BF in dogs of the CBMT group that presented higher rates of tumor proliferation and lower survival, indicating the participation of this adipocytokine in tumor aggressiveness. Moreover, to the best of our knowledge, this observation has not yet been reported in the veterinary oncology literature.

The evaluation of the performance indexes of serum resistin concentrations showed 75% co-positivity (sensitivity), 84.21% co-negativity (specificity), and a negative predictive value of 96.81%. Together, these indexes demonstrated that serum resistin concentrations can help to detect, in dogs with CBMT, the animals most likely to survive. Resistin concentrations have also been identified as an important prognostic factor in the progression of human breast cancer, which makes it a promising biomarker for breast tumor prognosis (18, 19).

We believe this is the first study in the evaluation of the serum concentrations of resistin in female dogs with CBMT and its relationship with the proliferative potential, obesity, and survival. The results indicate that hyperresistinemia is related to a higher tumor proliferative potential and excess body fat, which strengthens the proposal of its use as a prognostic marker in this frequent type of canine mammary cancer. In summary, female dogs with CBMT with higher weight, BMIc, and %BF showed higher resistin and lower survival rate. Hyperresistinemia was proportional to Ki-67 expression in the tumors of obese females, suggesting the participation of this adipocytokine in tumor aggressiveness.

## Data Availability Statement

All datasets generated for this study are included in the article/supplementary material.

## Ethics Statement

The animal study was reviewed and approved by Ethics Committee on the Use of Animals of the School of Veterinary Medicine of the Federal University of Bahia (protocol no. 05/2015) 307813/2018-5. Written informed consent was obtained from the owners for the participation of their animals in this study.

## Author Contributions

AE-L and BN designed the study. BN, CV-F, MM, and VB conducted the fieldwork. BN, MM, CV-F, KD, RP, and FS conducted the laboratory work. BN, TB, AE-L, SB-M, and RP wrote the manuscript. TB, MM, RP, EM-F, and AE-L revised the manuscript. AE-L was responsible for fund acquisition. All authors approved the final manuscript.

## Conflict of Interest

The authors declare that the research was conducted in the absence of any commercial or financial relationships that could be construed as a potential conflict of interest.
